# Building a more equitable Brazilian National Health System: 20 Years of Trans Visibility in Brazil

**DOI:** 10.1590/S2237-96222024v33e2024267.especial.en

**Published:** 2024-12-13

**Authors:** Alícia Krüger, Maria Amélia de Sousa Mascena Veras, Inês Dourado, Ethel Leonor Noia Maciel

**Affiliations:** 1 Ministério da Saúde, Secretaria de Vigilância em Saúde e Ambiente, Brasília, Distrito Federal, Brasil; 2 Faculdade de Ciências Médicas da Santa Casa de São Paulo, São Paulo, São Paulo, Brasil; 3 Universidade Federal da Bahia, Instituto de Saúde Coletiva, Salvador, Bahia, Brasil

The health of transgender people has received increasing attention in Brazil and globally, occupying various social spaces, with notable emphasis on scientific production that has seen substantial growth in recent years. A simple search on PubMed reveals that, between 2013 and 2023, scientific production on “transgender health” increased by almost 900%. Transgender people, by not conforming to society’s hegemonic norms and rules, are often discriminated against and excluded from various social spheres, such as family, school, work, as well as public and private spaces. They are often forced to live on the margins in their hometowns, migrate within their own country or even move abroad. This trajectory involves the continuous confrontation with stigmas and discrimination at multiple levels, including a high risk of gender-based violence, with physical and sexual assaults that deeply impact their physical and mental health.^1, 2, 3^

It is essential to acknowledge that the transgender population faces situations of extreme social vulnerability. In this context, the suffering caused by the daily experience of transphobia is exacerbated by the omission or restriction of health care — which is technically and scientifically viable today — by professionals and institutions.^4, 5, 6^

Two decades have passed since Trans Visibility began to gain momentum in Brazil, marking a unique and challenging trajectory, especially in the field of public health. During this period, the health sector has become the stage for intense struggles and significant achievements for the inclusion and guarantee of rights for transgender people. This movement, clearly led by transgender people themselves, reflects the power of social participation in health, a principle guaranteed since the establishment of the Brazilian National Health System (*Sistema Único de Saúde* – SUS).

Over the past 20 years, important achievements have been made, both in the health sector and in other areas of society. In the SUS, there has been recognition and use of social names, as well as the provision of specialized care for transgender people, such as hormone therapy, gender affirmation surgeries, and other body change interventions. Additionally, some health information and surveillance systems have incorporated fields for social names and gender identity. Outside the health sector, other significant victories have been achieved, such as the possibility of changing first names and sex in civil records, without requiring surgeries or medical reports, and the criminalization of homophobia and transphobia as a form of racism, as determined by the Supreme Federal Court.

This special issue of the Epidemiology and Health Services: Journal of the Brazilian National Health System (*Epidemiologia e Serviços de Saúde: revista do SUS* – RESS), celebrating 20 years of Trans Visibility in Brazil, aims to present articles that address different dimensions of the health and lives of trans people in the country. The Editorial Note, written by Simpson K. and Benevides B., reflects on the trajectory of these two decades of Trans Visibility, highlighting that, despite the intense stigma and high social vulnerability faced by this population, they have never been confined to these patterns or negative health outcomes. The leadership of transgender individuals, along with the commitment of various actors, demonstrates that active social participation translates into healthcare actions adapted to the specific needs of each population, in line with the principle of equity as provided for by the SUS.

The 29 articles comprising this issue were produced in different regions of Brazil, employing a variety of methodological approaches, and discuss access to health care, including prevention and prophylaxis (PrEP and vaccines), mental health, social vulnerabilities and violence, through original research, review studies and the aforementioned Editorial Note.

These articles address crucial issues related to the health, rights and living conditions of the transgender population in Brazil. They contribute to the understanding of the complex intersections between health, rights, violence, access to services and living conditions of the transgender population in Brazil.

We hope that the publication of these articles strengthens the commitment to an epidemiology and public health practice aimed at improving the living conditions of transgender people. Yes, it is about lives! We are talking about lives! This means not only identifying the specific needs of transgender people, but also promoting actions that improve their living conditions and health, based on scientific evidence and inclusive public policies. This is the commitment of the Secretariat of Health and Environmental Surveillance, the Ministry of Health and all those who acted as authors and editors. in this initiative.
