# Supplementary material for: Building a more equitable Brazilian National Health System: 20 Years of Trans Visibility in Brazil
Source: Epidemiol Serv Saude. 2024 Dec 13;33(spe1):e2024267. doi: 10.1590/S2237-96222024v33e2024267.especial.en (PMC11654811; doi:10.1590/S2237-96222024v33e2024267.especial.en)
Supplement: Translation

# Translation: A construção de um Sistema Único de Saúde mais equânime: 20 anos da Visibilidade Trans no Brasil

**DOI:** 10.1590/S2237-96222024v33e2024267.especial.pt

**Authors:** Alícia Krüger, Maria Amélia de Sousa Mascena Veras, Inês Dourado, Ethel Leonor Noia Maciel

**Affiliations:** 1 Ministério da Saúde, Secretaria de Vigilância em Saúde e Ambiente, Brasília, Distrito Federal, Brasil; 2 Faculdade de Ciências Médicas da Santa Casa de São Paulo, São Paulo, São Paulo, Brasil; 3 Universidade Federal da Bahia, Instituto de Saúde Coletiva, Salvador, Bahia, Brasil

A saúde de pessoas trans tem recebido crescente atenção no Brasil e no mundo, ocupando diversos espaços sociais, com destaque para a produção científica, que teve um crescimento substancial nos últimos anos. Uma simples busca no PubMed revela que, entre 2013 e 2023, a produção científica sobre “*transgender health*” aumentou quase 900%. Pessoas trans, por não se enquadrarem nas normas e regras hegemônicas da sociedade, são frequentemente discriminadas e excluídas de diversas esferas de socialização, como a família, a escola, o trabalho, e espaços públicos e privados. Muitas vezes, são forçadas a viver à margem em suas cidades de origem, migrar dentro do próprio país ou até mesmo para o exterior. Essa trajetória envolve o enfrentamento contínuo de estigmas e discriminações em diferentes níveis, incluindo um risco elevado de violência de gênero, com agressões físicas e sexuais que impactam profundamente sua saúde física e mental.^1,2,3^

É fundamental reconhecer que a população trans enfrenta situações de extrema vulnerabilidade social. Nesse contexto, o sofrimento decorrente da transfobia vivida diariamente é agravado pela omissão ou restrição do cuidado em saúde – hoje viável em termos biotecnocientíficos – por parte de profissionais e instituições.^4,5,6^

Duas décadas se passaram desde que a Visibilidade Trans começou a ganhar força no Brasil, marcando uma trajetória única e desafiadora, especialmente no campo da saúde coletiva. Ao longo desse período, o setor da saúde se tornou palco de intensas lutas e importantes conquistas pela inclusão e garantia de direitos para as pessoas trans. Esse movimento, claramente protagonizado pelas próprias pessoas trans, reflete a potência da participação social na saúde, princípio garantido desde a criação do Sistema Único de Saúde (SUS).

Ao longo desses 20 anos, importantes conquistas foram alcançadas, tanto na área da saúde quanto em outras esferas da sociedade. No SUS, houve o reconhecimento e a utilização do nome social, além da oferta de cuidados especializados para pessoas trans, como hormonização, cirurgias de redesignação sexual e outras intervenções de modificação corporal. Também foram incluídos, em alguns sistemas de informação e vigilância em saúde, os campos de nome social e identidade de gênero. Fora do setor da saúde, outras vitórias significativas foram obtidas, como a possibilidade de alteração de prenome e sexo nos registros civis, sem a exigência de cirurgias ou laudos médicos, e a criminalização da homotransfobia como crime de racismo, conforme decisão do Supremo Tribunal Federal.

Este número especial da *Revista Epidemiologia e Serviços de Saúde*: revista do SUS (RESS), em comemoração aos 20 anos da Visibilidade Trans no Brasil, tem como objetivo apresentar artigos que abordam diferentes dimensões da saúde e da vida de pessoas trans no país. A nota editorial, escrita por Simpson K. e Benevides B., reflete sobre a trajetória dessas duas décadas de Visibilidade Trans, destacando que, apesar do intenso estigma e da alta vulnerabilidade social, essa população nunca se limitou a esses padrões ou aos desfechos negativos em saúde. O protagonismo das pessoas trans, aliado ao comprometimento de diversos atores e atrizes, demonstra que a participação social ativa se traduz em ações de saúde adaptadas às necessidades específicas de cada população, em conformidade com o princípio da equidade previsto pelo SUS.

Os 29 artigos que compõem este número foram produzidos em diferentes regiões do Brasil, empregando uma variedade de abordagens metodológicas, e discutem o acesso a cuidados de saúde, incluindo prevenção e profilaxias (PrEP e vacinas), saúde mental, vulnerabilidades sociais e violências, por meio de pesquisas originais, e estudos de revisão.

Esses artigos tratam de questões fundamentais envolvendo saúde, direitos e condições de vida da população trans no Brasil, além de oferecerem uma análise das complexas interseções entre esses temas, incluindo violência e acesso a serviços essenciais.

Esperamos que a publicação desses artigos contribua para fortalecer o compromisso de uma epidemiologia e de uma saúde coletiva voltadas para a melhoria das condições de vida das pessoas trans. Sim, trata-se de vidas! Ou seja, não apenas identificar as necessidades específicas das pessoas trans, mas também promover ações que melhorem suas condições de vida e saúde, com base em evidências científicas e políticas públicas inclusivas. Esse é o compromisso da Secretaria de Vigilância em Saúde e Ambiente do Ministério da Saúde e de todas as pessoas que atuaram como autoras e editoras nesta iniciativa.
